# Supplementary material for: A yeast phenomic model for the influence of Warburg metabolism on genetic buffering of doxorubicin
Source: Cancer Metab. 2019 Oct 23;7:9. doi: 10.1186/s40170-019-0201-3 (PMC6806529; doi:10.1186/s40170-019-0201-3)
Supplement: Supplementary file 9 — Additional file 9. GO term-specific heatmaps for REMc/GTF-enriched clusters. GO term-specific heatmaps for significant GO process terms were generated as described in methods and Figs. 3 and 4. Any related child terms are presented in subsequent pages of the parent file name. GO terms with more than 100 children, with 2 or fewer genes annotated to the term, or a file size over 300KB are not shown. All heatmaps are generated with the same layout (see Figs. 3 and 4). [file 40170_2019_201_MOESM9_ESM.bz2 › Additional_File9_GOTermHeatmaps/Additional_File9_GOTermHeatmap/protein_transmembrane_transport.pdf]

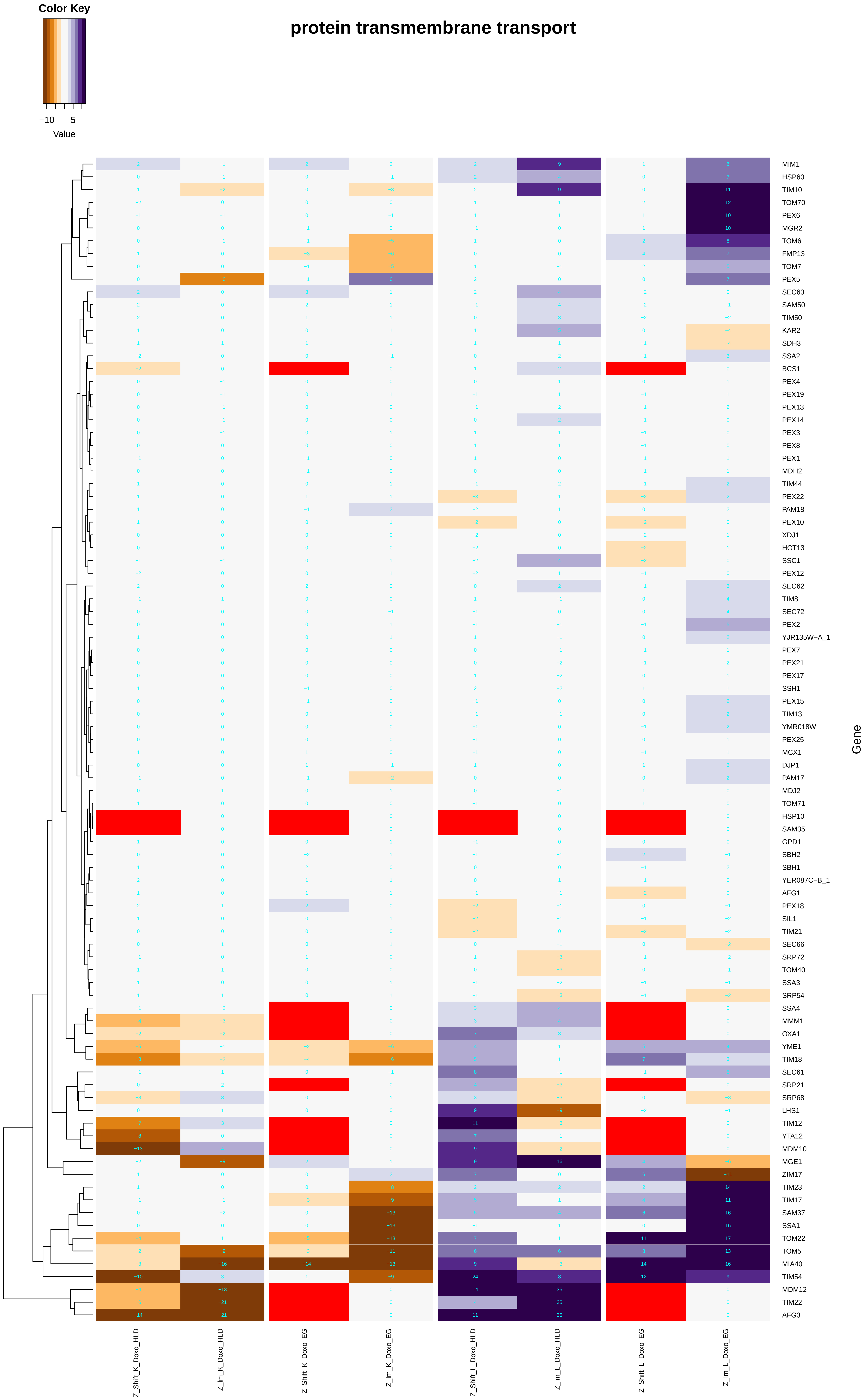

protein transmembrane transport
Color Key
−10
5
Value
MIM1
HSP60
TIM10
TOM70
PEX6
MGR2
TOM6
FMP13
TOM7
PEX5
SEC63
SAM50
TIM50
KAR2
SDH3
SSA2
BCS1
PEX4
PEX19
PEX13
PEX14
PEX3
PEX8
PEX1
MDH2
TIM44
PEX22
PAM18
PEX10
XDJ1
HOT13
SSC1
PEX12
SEC62
TIM8
SEC72
PEX2
YJR135W−A_1
PEX7
PEX21
PEX17
SSH1
PEX15
TIM13
YMR018W
PEX25
MCX1
DJP1
PAM17
MDJ2
TOM71
HSP10
SAM35
GPD1
SBH2
SBH1
YER087C−B_1
AFG1
PEX18
SIL1
TIM21
SEC66
SRP72
TOM40
SSA3
SRP54
SSA4
MMM1
OXA1
YME1
TIM18
SEC61
SRP21
SRP68
LHS1
TIM12
YTA12
MDM10
MGE1
ZIM17
TIM23
TIM17
SAM37
SSA1
TOM22
TOM5
MIA40
TIM54
MDM12
TIM22
AFG3
Gene
Z_Shift_K_Doxo_HLD
Z_lm_K_Doxo_HLD
Z_Shift_K_Doxo_EG
Z_lm_K_Doxo_EG
Z_Shift_L_Doxo_HLD
Z_lm_L_Doxo_HLD
Z_Shift_L_Doxo_EG
Z_lm_L_Doxo_EG

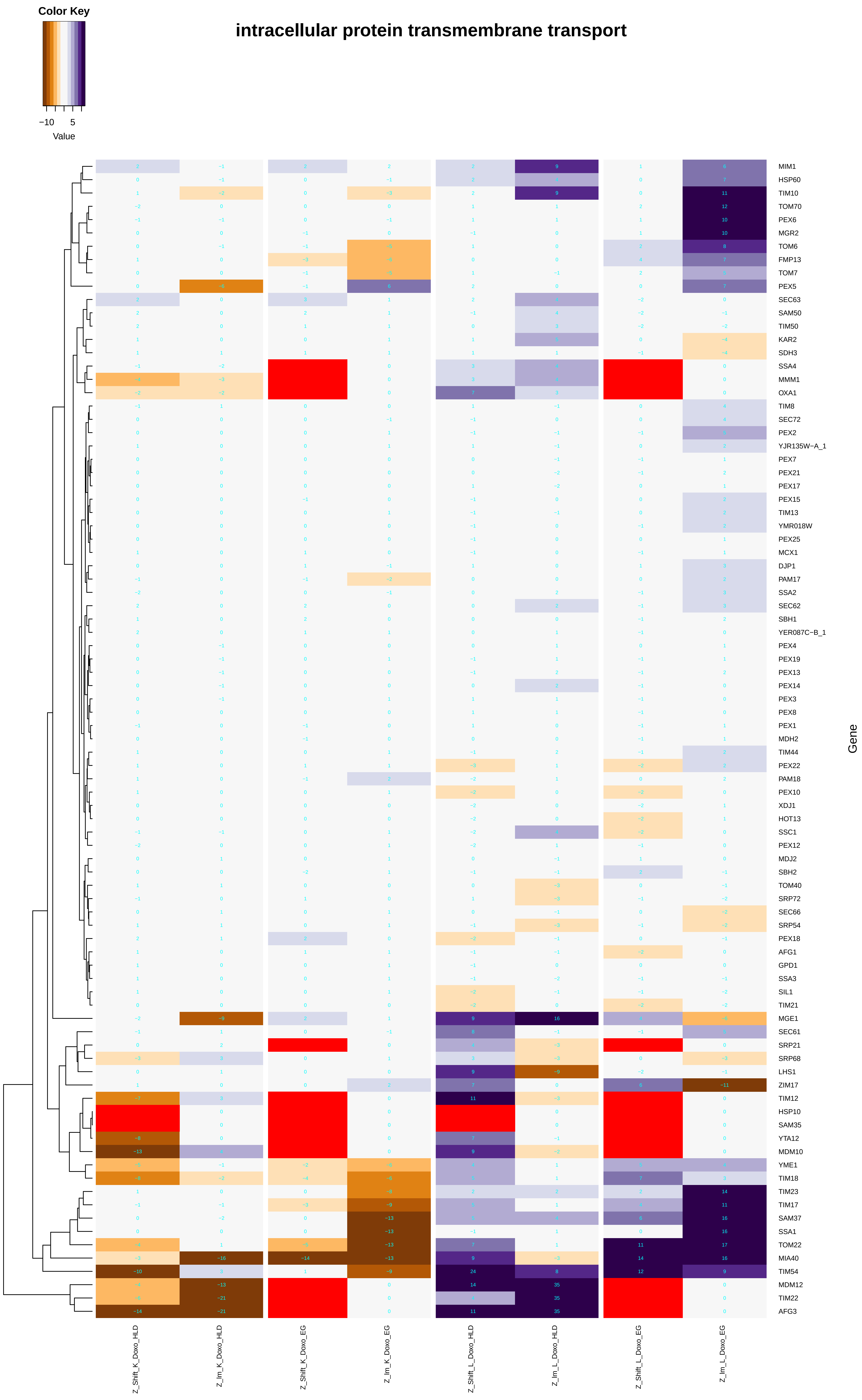

intracellular protein transmembrane transport
Color Key
−10
5
Value
MIM1
HSP60
TIM10
TOM70
PEX6
MGR2
TOM6
FMP13
TOM7
PEX5
SEC63
SAM50
TIM50
KAR2
SDH3
SSA4
MMM1
OXA1
TIM8
SEC72
PEX2
YJR135W−A_1
PEX7
PEX21
PEX17
PEX15
TIM13
YMR018W
PEX25
MCX1
DJP1
PAM17
SSA2
SEC62
SBH1
YER087C−B_1
PEX4
PEX19
PEX13
PEX14
PEX3
PEX8
PEX1
MDH2
TIM44
PEX22
PAM18
PEX10
XDJ1
HOT13
SSC1
PEX12
MDJ2
SBH2
TOM40
SRP72
SEC66
SRP54
PEX18
AFG1
GPD1
SSA3
SIL1
TIM21
MGE1
SEC61
SRP21
SRP68
LHS1
ZIM17
TIM12
HSP10
SAM35
YTA12
MDM10
YME1
TIM18
TIM23
TIM17
SAM37
SSA1
TOM22
MIA40
TIM54
MDM12
TIM22
AFG3
Gene
Z_Shift_K_Doxo_HLD
Z_lm_K_Doxo_HLD
Z_Shift_K_Doxo_EG
Z_lm_K_Doxo_EG
Z_Shift_L_Doxo_HLD
Z_lm_L_Doxo_HLD
Z_Shift_L_Doxo_EG
Z_lm_L_Doxo_EG

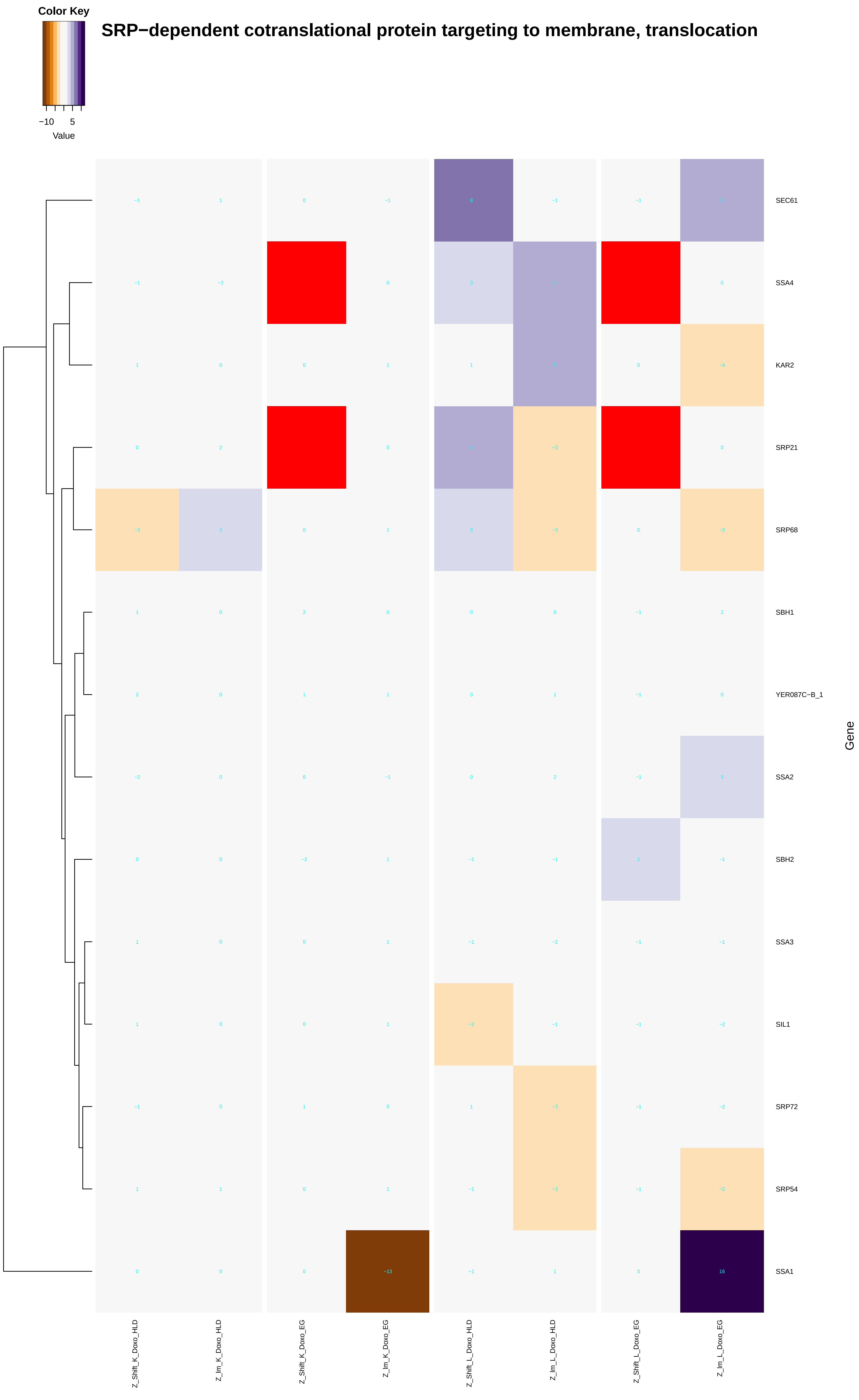

Color Key
−10
5
Value
SRP−dependent cotranslational protein targeting to membrane, translocation
SEC61
SSA4
KAR2
SRP21
SRP68
SBH1
YER087C−B_1
SSA2
SBH2
SSA3
SIL1
SRP72
SRP54
SSA1
Gene
Z_Shift_K_Doxo_HLD
Z_Im_K_Doxo_HLD
Z_Shift_K_Doxo_EG
Z_Im_K_Doxo_EG
Z_Shift_L_Doxo_HLD
Z_Im_L_Doxo_HLD
Z_Shift_L_Doxo_EG
Z_Im_L_Doxo_EG

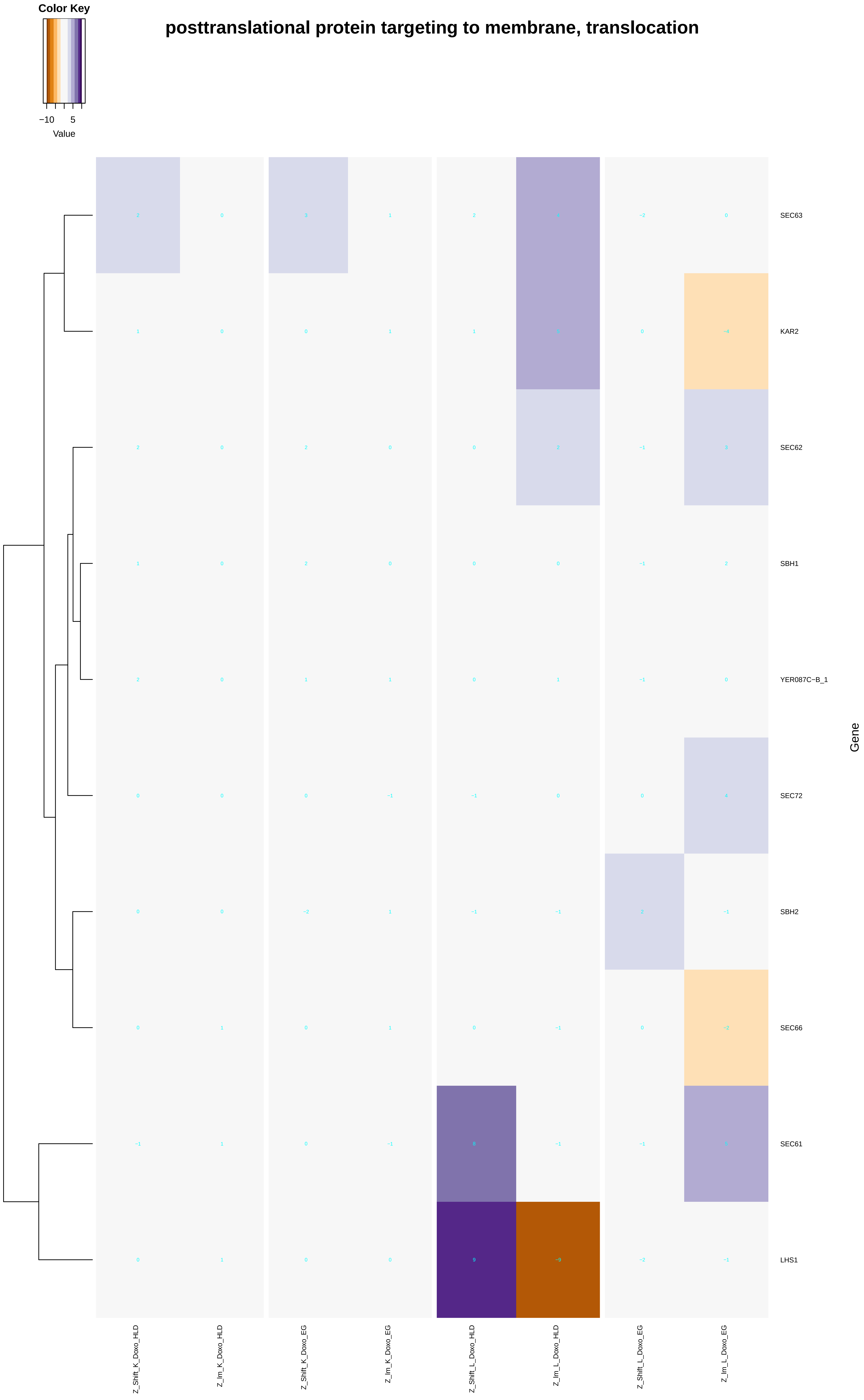

Color Key
−10
5
Value
posttranslational protein targeting to membrane, translocation
SEC63
KAR2
SEC62
SBH1
YER087C−B_1
SEC72
SBH2
SEC66
SEC61
LHS1
Gene
Z_Shift_K_Doxo_HLD
Z_lm_K_Doxo_HLD
Z_Shift_K_Doxo_EG
Z_lm_K_Doxo_EG
Z_Shift_L_Doxo_HLD
Z_lm_L_Doxo_HLD
Z_Shift_L_Doxo_EG
Z_lm_L_Doxo_EG

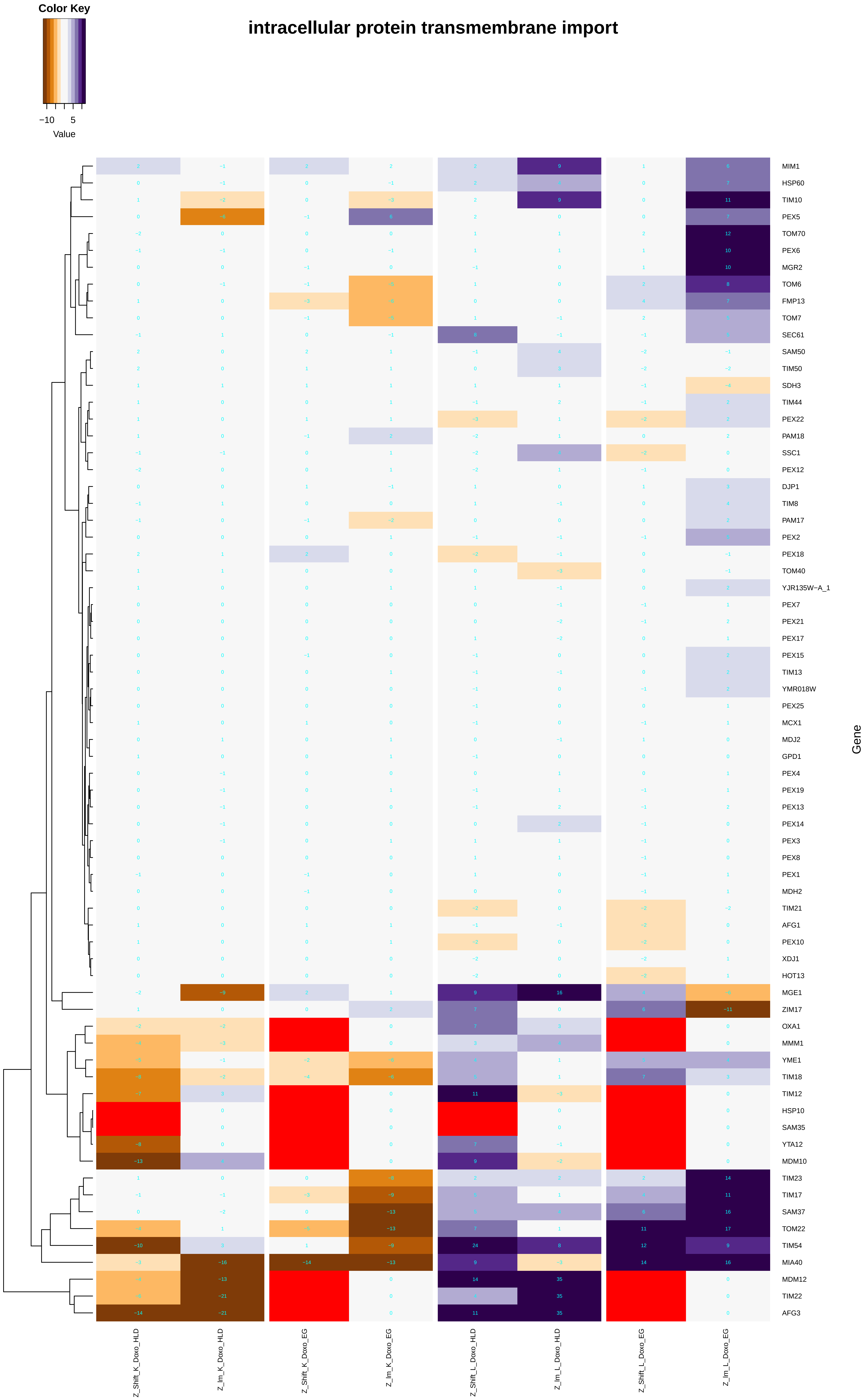
intracellular protein transmembrane import
Color Key
−10
5
Value
MIM1
HSP60
TIM10
PEX5
TOM70
PEX6
MGR2
TOM6
FMP13
TOM7
SEC61
SAM50
TIM50
SDH3
TIM44
PEX22
PAM18
SSC1
PEX12
DJP1
TIM8
PAM17
PEX2
PEX18
TOM40
YJR135W−A_1
PEX7
PEX21
PEX17
PEX15
TIM13
YMR018W
PEX25
MCX1
MDJ2
GPD1
PEX4
PEX19
PEX13
PEX14
PEX3
PEX8
PEX1
MDH2
TIM21
AFG1
PEX10
XDJ1
HOT13
MGE1
ZIM17
OXA1
MMM1
YME1
TIM18
TIM12
HSP10
SAM35
YTA12
MDM10
TIM23
TIM17
SAM37
TOM22
TIM54
MIA40
MDM12
TIM22
AFG3
Gene
Z_Shift_K_Doxo_HLD
Z_Im_K_Doxo_HLD
Z_Shift_K_Doxo_EG
Z_Im_K_Doxo_EG
Z_Shift_L_Doxo_HLD
Z_Im_L_Doxo_HLD
Z_Shift_L_Doxo_EG
Z_Im_L_Doxo_EG

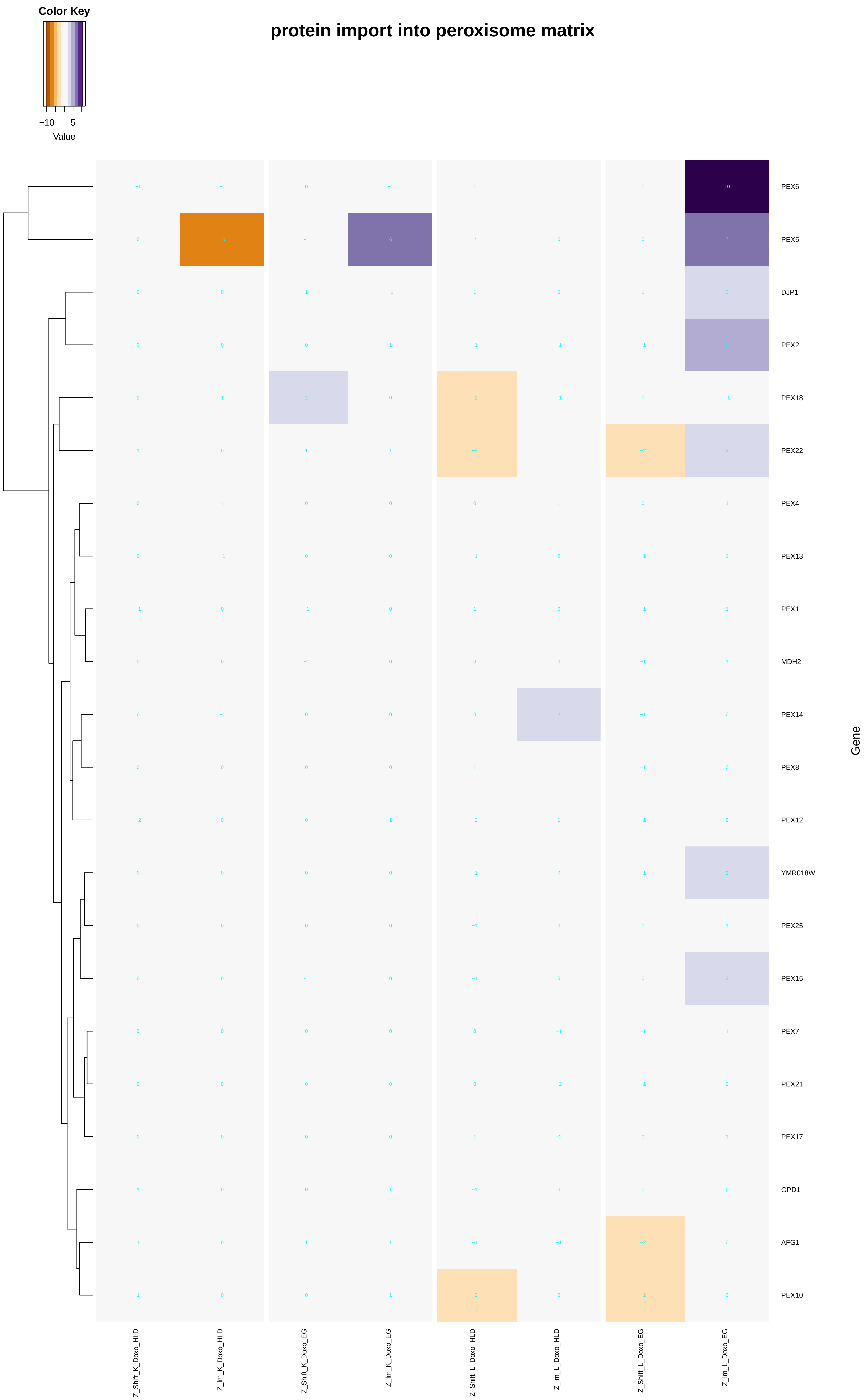
Color Key
−10
5
Value
protein import into peroxisome matrix
PEX6
PEX5
DJP1
PEX2
PEX18
PEX22
PEX4
PEX13
PEX1
MDH2
PEX14
PEX8
PEX12
YMR018W
PEX25
PEX15
PEX7
PEX21
PEX17
GPD1
AFG1
PEX10
Gene
Z_Shift_K_Doxo_HLD
Z_lm_K_Doxo_HLD
Z_Shift_K_Doxo_EG
Z_lm_K_Doxo_EG
Z_Shift_L_Doxo_HLD
Z_lm_L_Doxo_HLD
Z_Shift_L_Doxo_EG
Z_lm_L_Doxo_EG

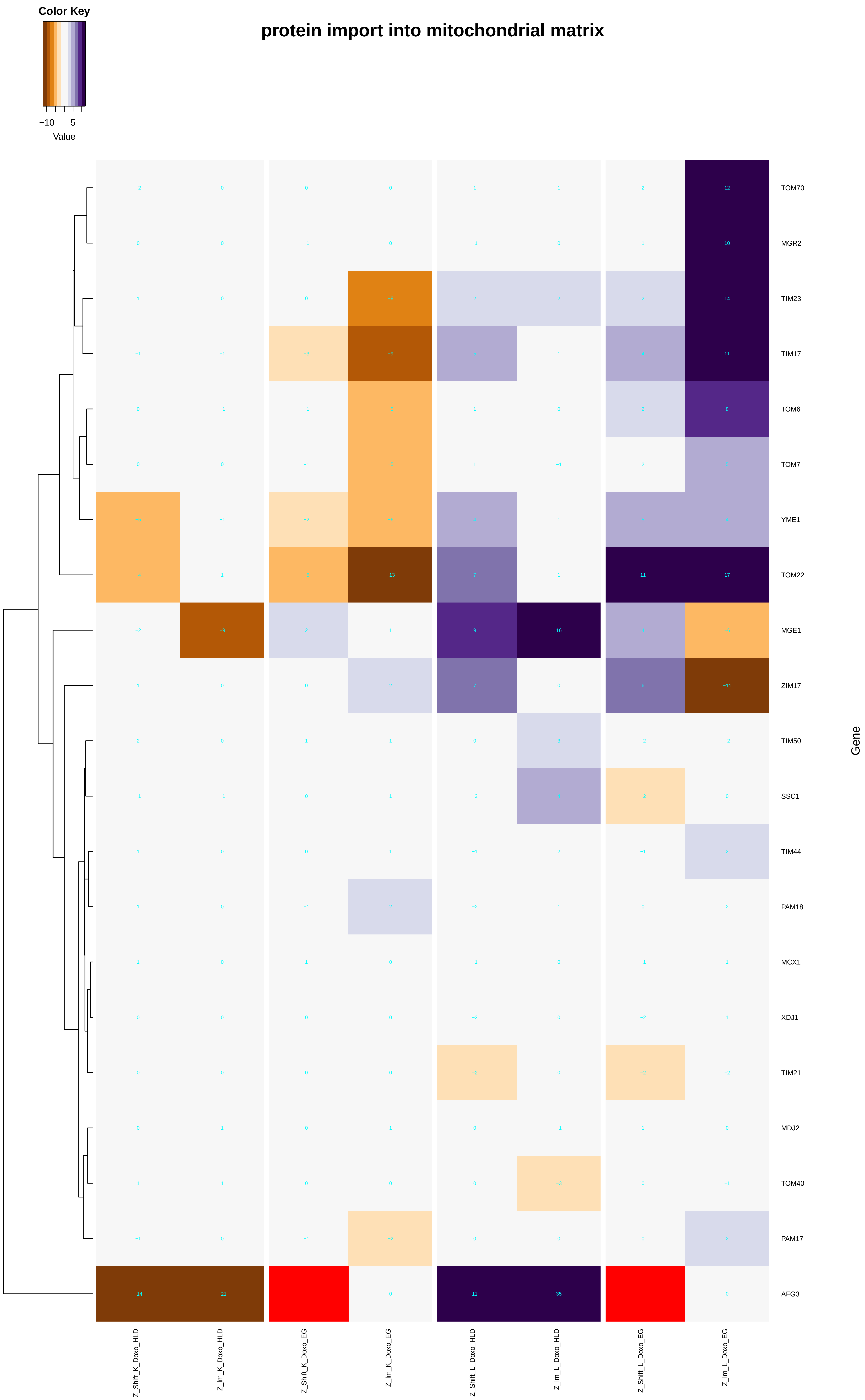
protein import into mitochondrial matrix
Color Key
−10
5
Value
TOM70
MGR2
TIM23
TIM17
TOM6
TOM7
YME1
TOM22
MGE1
ZIM17
TIM50
SSC1
TIM44
PAM18
MCX1
XDJ1
TIM21
MDJ2
TOM40
PAM17
AFG3
Gene
Z_Shift_K_Doxo_HLD
Z_Im_K_Doxo_HLD
Z_Shift_K_Doxo_EG
Z_Im_K_Doxo_EG
Z_Shift_L_Doxo_HLD
Z_Im_L_Doxo_HLD
Z_Shift_L_Doxo_EG
Z_Im_L_Doxo_EG

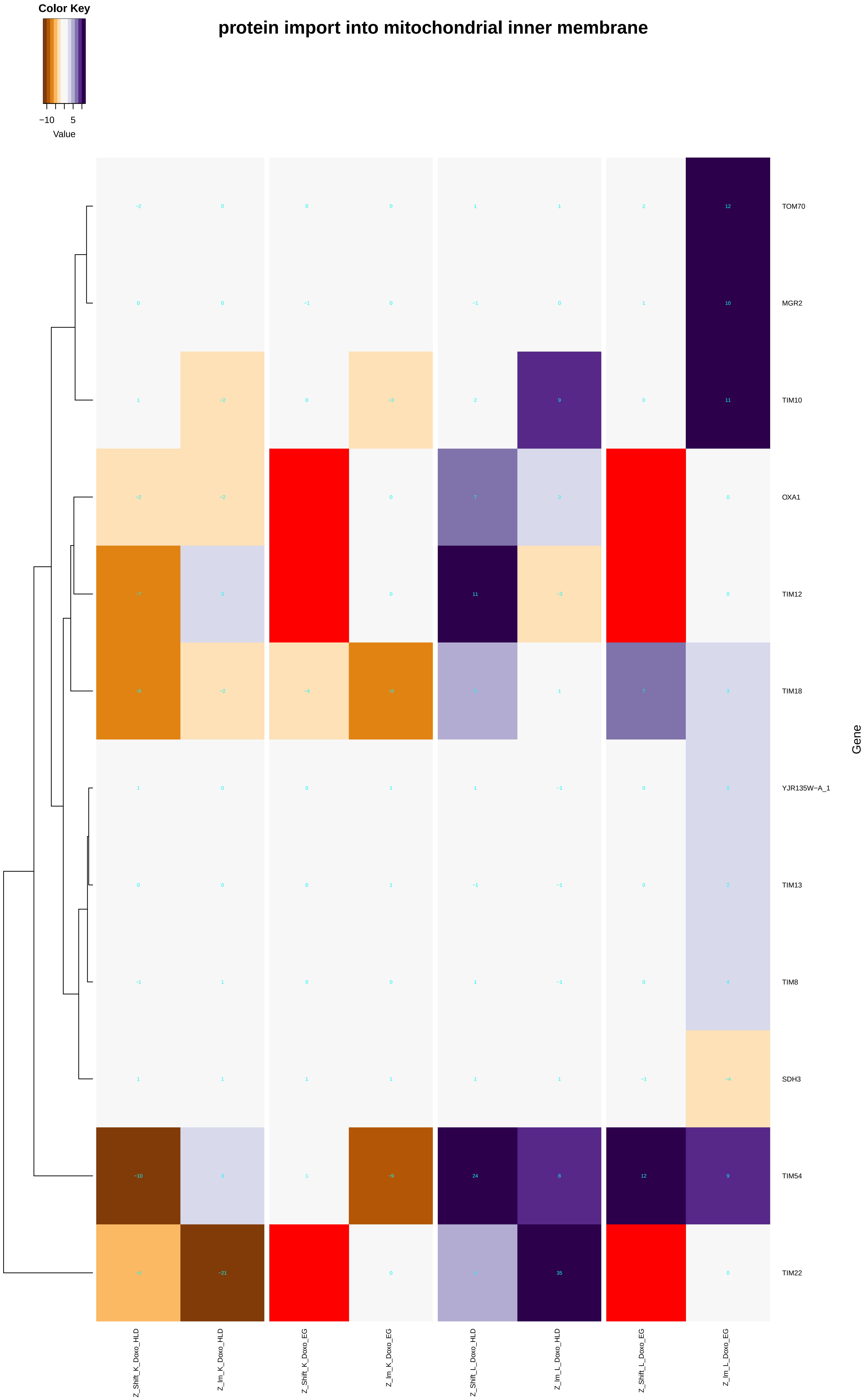

Color Key
−10
5
Value
protein import into mitochondrial inner membrane
TOM70
MGR2
TIM10
OXA1
TIM12
TIM18
YJR135W−A_1
TIM13
TIM8
SDH3
TIM54
TIM22
Gene
Z_Shift_K_Doxo_HLD
Z_Im_K_Doxo_HLD
Z_Shift_K_Doxo_EG
Z_Im_K_Doxo_EG
Z_Shift_L_Doxo_HLD
Z_Im_L_Doxo_HLD
Z_Shift_L_Doxo_EG
Z_Im_L_Doxo_EG

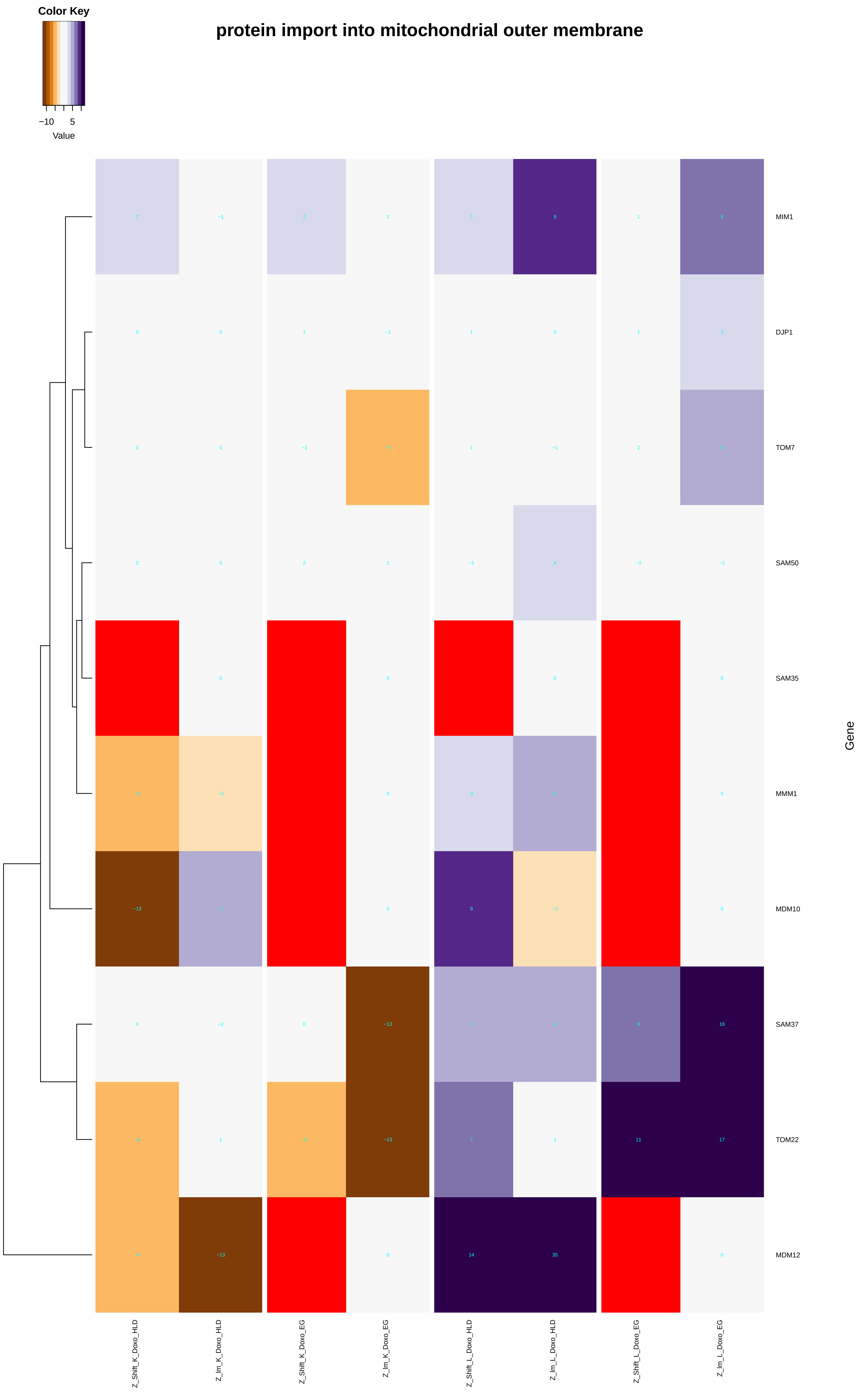

Color Key
−10
5
Value
protein import into mitochondrial outer membrane
MIM1
DJP1
TOM7
SAM50
SAM35
MMM1
MDM10
SAM37
TOM22
MDM12
Gene
Z_Shift_K_Doxo_HLD
Z_Im_K_Doxo_HLD
Z_Shift_K_Doxo_EG
Z_Im_K_Doxo_EG
Z_Shift_L_Doxo_HLD
Z_Im_L_Doxo_HLD
Z_Shift_L_Doxo_EG
Z_Im_L_Doxo_EG

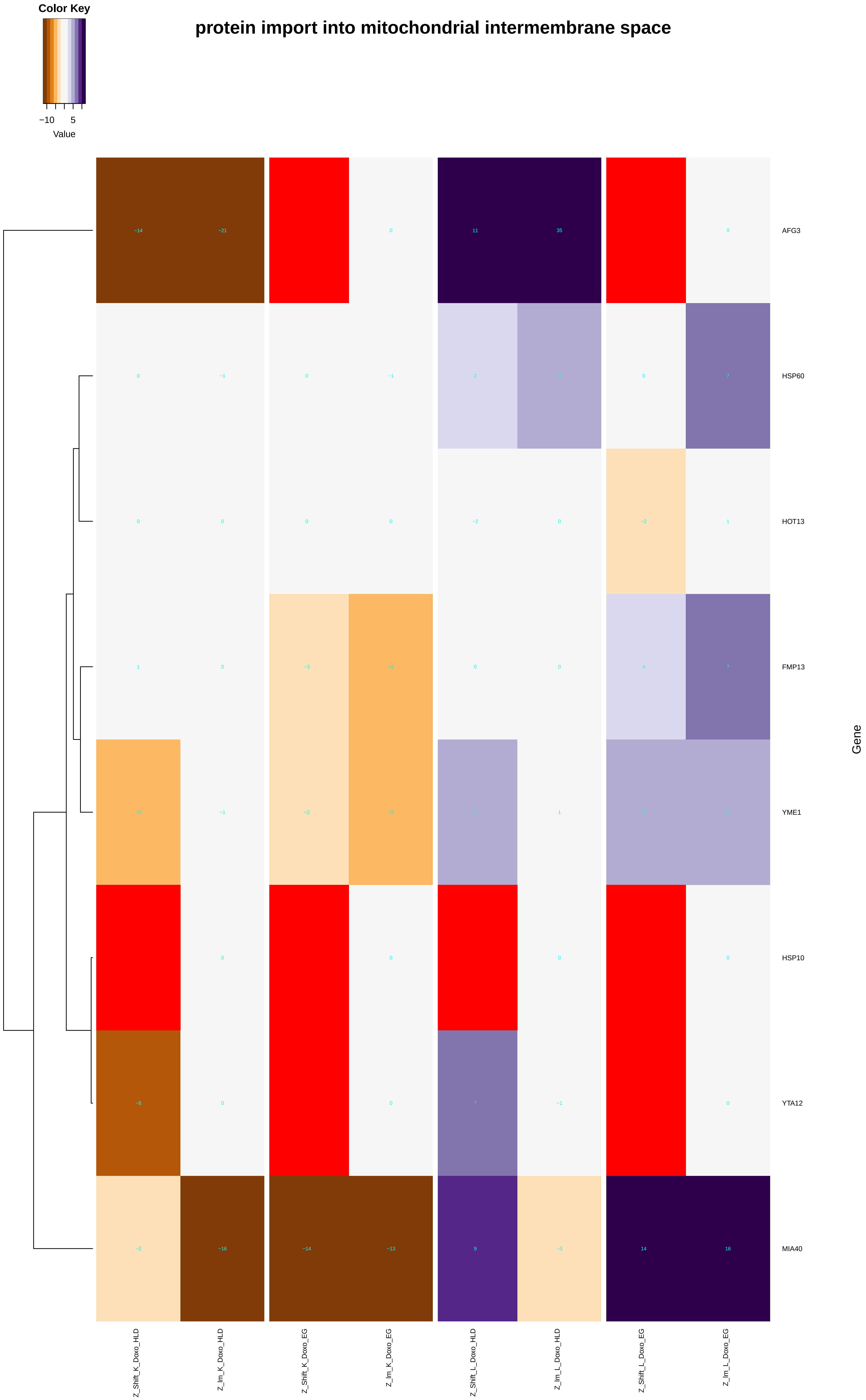

protein import into mitochondrial intermembrane space
Color Key
−10
5
Value
AFG3
HSP60
HOT13
FMP13
YME1
HSP10
YTA12
MIA40
Gene
Z_Shift_K_Doxo_HLD
Z_Im_K_Doxo_HLD
Z_Shift_K_Doxo_EG
Z_Im_K_Doxo_EG
Z_Shift_L_Doxo_HLD
Z_Im_L_Doxo_HLD
Z_Shift_L_Doxo_EG
Z_Im_L_Doxo_EG
